# Neuroprotective effects of* Tiliacora triandra *leaf extract in a mice model of cerebral ischemia reperfusion 

**Published:** 2020

**Authors:** Wachiryah Thong-asa, Vasakorn Bullangpoti

**Affiliations:** 1 *Animal Toxicology and Physiology Specialty Research Unit (ATPSRU), Physiology Division, Department of Zoology, Faculty of Science, Kasetsart University, Bangkok, Thailand*

**Keywords:** Tiliacora triandra, Ischemic-reperfusion injury, Bilateral common carotid artery occlusion, Cerebral cortex, Hippocampus, Lipid peroxidation

## Abstract

**Objective:**

The present study investigated possible neuroprotective effects of ethanolic extract of *Tiliacora triandra* leaf against cerebral ischemic-reperfusion injury in mice.

**Materials and Methods:**

Forty male Institute of Cancer Research (ICR) mice were randomly divided into five groups: (1) Sham + 10% Tween 80, (2) bilateral common carotid artery occlusion (BCCAO) + 10% Tween 80, (3) BCCAO + *T. triandra* 300 mg/kg, (4) BCCAO + *T. triandra* 600 mg/kg and (5) BCCAO + quercetin 10 mg/kg. Cerebral ischemic-reperfusion (IR) was induced by 30 min of BCCAO followed by 45 min of reperfusion. After IR induction, total brain protein, calcium, malondialdehyde (MDA), catalase (CAT), superoxide dismutase (SOD), and reduced glutathione (GSH), as well as brain infraction and histopathological changes in vulnerable brain areas, such as the cerebral cortex and hippocampus, were evaluated.

**Results:**

The results showed that 2 weeks of pretreatment with *T. triandra* leaf extract at doses of 300 and 600 mg/kg significantly reduced calcium and MDA, but increased GSH and SOD and CAT activities. The extract significantly attenuated brain infarction and neuronal death in the cerebral cortex and hippocampus.

**Conclusion:**

We demonstrated the neuroprotective effects of *T. triandra* leaf extract against cerebral IR injury in mice.

## Introduction

Cerebral ischemic-reperfusion (IR) injury following brain transient ischemia occurs in stroke and cardiac arrest. Multiple neuronal death-promoting pathomechanisms, such as cytosolic calcium overload, excitotoxicity, free radical formation, inflammation, protein synthesis inhibition and subsequent necrotic and apoptotic cell death, occur during IR   (White et al., 2000 ▶) . During an ischemic event, survival-promoting mechanisms, such as the formation of heat shock proteins, anti-inflammatory cytokines, growth factors and antioxidants, are activated (Leker and Shohami, 2002 ▶) . The balance between death- and survival-promoting mechanisms during IR depends on the severity and duration of the ischemia. Moreover, during the reperfusion period, high blood oxygen levels promote free radical formation and exacerbate neuronal death   (White et al., 2000 ▶) . Inhibiting death-promoting mechanisms while enhancing survival-promoting mechanisms is a neuroprotective therapeutic ideal that may increase neuronal endurance and reduce disability in patients who suffer from IR injuries. 

Neuroprotective therapy has recently gained attention. Its objective is to reduce neuronal vulnerability to ischemia and extend the effective therapeutic window for thrombolytic reperfusion injuries. Many synthetic antioxidant chemicals are used to diminish oxidative stress in cerebral IR injuries, and most such chemicals are polyphenolic and flavonoid compounds with free-radical scavenging properties  (Makarov et al., 2005 ▶; Mira et al., 2002 ▶; Pedrielli et al., 2001 ▶) . These compounds are of natural origin and function as phytomedicines. 

*Tiliacora triandra* (Colebr.) Diels is a plant of the Menispermaceae family that is native to Southeast Asia. It is frequently used in northeastern Thai cuisine as well as traditional folk medicine for its anti-pyretic, anti-bacterial and anti-malarial properties, and for alcoholic detoxification  (Pachaly and Khosravian, 1988 ▶; Paris and Sasorith, 1967 ▶; Saiin and Markmee, 2003 ▶; Sureram et al., 2012 ▶) . It is also used for its anti-inflammatory, anti-cancer, acetylcholine esterase inhibitory and antioxidant properties  (Ingkaninan et al., 2003 ▶; Kaewpiboon et al., 2014 ▶; Phadungkit et al., 2012 ▶) . These properties were reported along with high concentrations of polyphenolic and flavonoid compounds  (Boonsong et al., 2009 ▶; Singthong et al., 2014 ▶) . *T. triandra* is a natural source of antioxidants because it contains high levels of beta-carotene, condensed tannins, triterpene, flavonoids, saponin, phytol and alpha-tocopherol (Boonsong et al., 2009 ▶) . Acute and subchronic toxicity studies of *T. triandra *leaf extract in rats showed no toxicity signs when rats were given a single dose of 5,000 mg/kg or up to 1,200 mg/kg continuously for 90 days (Sireeratawong et al., 2008 ▶) . The neuroprotective and neuronal promoting effects of *T. triandra* leaf extract were also reported (Thong-asa et al., 2017 ▶). Regarding the extract’s antioxidant properties, which may ameliorate the major pathomechanism of IR injury, the present study was aimed at investigating the neuroprotective effects of *T. triandra* leaf extract against cerebral IR injury in mice. 

## Materials and Methods

**Animals**

Animal care and experimental protocols were approved by the Animal Ethic Committee, Kasetsart University Research and Development Institute (KURDI), Kasetsart University (ID# ACKU 02756). Forty male ICR mice were obtained from the National Laboratory Animal Center (NLAC), Mahidol University, Salaya, Nakornprathom. They were housed in a room with well-controlled temperature and humidity, with 12-hr light and dark periods and provided with standard pellet food and reverse osmosis (RO) water *ad libitum*. 

***T. triandra*** **leaf extract**

Ethanolic extract of *T. triandra *leaves was obtained from the Animal Toxicology and Physiology Specialty Research Unit (ATPSRU). Air-dried *T. triandra* leaves were powdered and extracted using 95% ethanol in a Soxhlet extractor for 18–20 hr. The extract was then filtered and concentrated using a rotary vacuum. The extract’s flavonoid and phenol contents were 231.29 mg QE/g and 340.21 mg GAE/g, respectively (Thong-asa et al., 2017 ▶). The extract was diluted in 10% Tween 80 before use. 

**Experimental protocol**

Mice were randomly divided into 5 groups: (1) Sham + 10% Tween 80, (2) BCCAO + 10% Tween 80, (3) BCCAO + *T. triandra* 300 mg/kg, (4) BCCAO + *T. triandra* 600 mg/kg and (5) BCCAO + quercetin 10 mg/kg. Gavage administration was done for 2 weeks prior to cerebral IR induction to verify the neuroprotective effects of pre-ischemic *T. triandra* treatment (Vaghef and Bafandeh Gharamaleki, 2017 ▶). Cerebral IR was induced by 30 min of BCCAO followed by 45 min of reperfusion (Raghavendra et al., 2009; Sakamula and Thong-Asa, 2018). After IR, all animals were decapitated, and their brains were collected for biochemical and histological analysis. The brains were washed in cold 0.9% normal saline solution (NSS) and homogenized in 10% w/v 0.05 M phosphate buffered saline (PBS, pH 7.4). Supernatant was prepared by centrifugation of homogenate 10,000g at 4^o^C. 

**Total protein determination**

We mixed 0.2 ml of supernatant with 2 ml of solution D (2% w/v Na_2_CO_3_ in 0.1 N NaOH: 0.5% w/v CuSO_4_-5H_2_O in distilled water: 1% w/v C_4_H_4_KNaO_6_-4H_2_O (48:1:1)). We then incubated the mixture for 10 min and added 0.2 ml of 1 N Folin-Ciocalteu reagent (1:1). After 30 min of incubation, the absorbance of the mixture was read at 600 nm (Lowry et al., 1951 ▶). Protein concentration was calculated using a standard curve (y=5.1314x+0.0249, r^2^=0.9916) prepared from bovine serum albumin at concentrations of 0, 0.083, 0.153, 0.214 and 0.266 mg/ml. 

**Calcium determination**

Blank (2.55 ml of distilled water + 1.5 ml of working color reagent), standard (2.5 ml of distilled water + 0.05 ml working standard calcium solution + 1.5 ml of working color reagent) and test (2.5 ml of distilled water + 0.05 ml of sample + 1.5 ml of working color reagent) mixtures were prepared. The mixtures were incubated at 25°C for 5 min, then read at 490 nm reference against a blank. Calcium concentration was interpreted as mEq/L (Spare, 1964 ▶).

**Malondialdehyde (MDA) determination**

We mixed 0.2 ml of homogenate with 0.2 ml of 4% sodium dodecyl sulfate, 1.5 ml of 20% acetic acid and 1.5 ml of 0.5% thiobarbituric acid, and boiled them for 60 min at 95°C. The mixture was centrifuged for 10 min (3,500 rpm), and the absorbance of the supernatant was read at 532 nm. The MDA concentration was interpreted as µmoles/mg of protein using the standard curve (y=0.0057x+0.0547, r^2^=0.9907) (Sakamula and Thong-Asa, 2018 ▶).

**Superoxide dismutase (SOD) determination**

We mixed 0.1 ml of supernatant with 0.1 ml of EDTA (1×10^-4^ M), 0.5 ml of carbonate buffer (pH 7.9) and 1 ml of epinephrine (3×10^-3^ M). The absorbance of the mixture was read at 480 nm every 30 sec for 3 min. Enzyme activity was interpreted as U/min/mg of protein using the standard curve (y=0.0015x+0.0001, r^2^=0.998) plotted for SOD concentration that included 0, 0.0058, 0.0294, 0.117 and 0.294 µg/mg (standard SOD activity was 6150 U/mg, Merck, Germany) (Sakamula and Thong-Asa, 2018 ▶).

**Catalase (CAT) determination**

Here, 50 µl of supernatant was taken and the volume was made up to 3 ml with 0.05 M PBS (pH 7.4) containing 0.01 M of H_2_O_2_. The absorbance of the mixture was read continuously at 240 nm every 30 sec for 3 min. CAT level was calculated with reference to the extinction coefficient of H_2_O_2_ and interpreted as µmoles of H_2_O_2 _utilized/min/mg of protein (U/mg of protein) (Hadwan and Abed, 2016 ▶). 

**Reduced glutathione (GSH) determination**

We mixed 1 ml of homogenate with 1 ml of 10% tricarboxylic acid (TCA) and centrifuged the mixture. Thereafter, 0.5 ml of the supernatant was mixed with 2 ml of 5, 5’-dithios 2-nitro benzoic acid. The volume was increased to 3 ml with PBS, and the mixture was read at 412 nm. A standard curve (y=0.5817x–0.0227, r^2^=0.993) of glutathione was prepared using concentrations of 0, 0.065, 0.163, 0.326, 0.490 and 0.653 mM and GSH was represented as mmoles/mg of protein (Sakamula and Thong-Asa, 2018 ▶). 

**Infarction area determination**

Brains were removed quickly after decapitation, briefly washed in cold 0.9% NSS and cut by a surgical blade to yield 2 mm of thickness using an acrylic brain template. Brain pieces were stained with 2% 2, 3, 5-triphenyltetrazolium chloride at 37°C for 10 min. After staining, brain pieces were kept in 10% neutral buffer formalin for 24 hr and captured for infarction analysis using NIH Image J. 

**Histopathological analysis**

We performed histological analysis by staining with 0.1% Luxol fast blue and 0.1% cresyl violet. Brains were embedded in paraffin and cut to a thickness of 5 µm. Five slides were selected from each animal starting at -1.98 from bregma (Paxinos and Franklin, 2008 ▶) with an interval of 100 µm. All selected slides were incubated overnight in hot air oven at 60°C. Brain slides were deparaffinized and rehydrated using serial dilutions of xylene, 100% ethanol (EtOH) and 95% EtOH, and soaked in 0.1% Luxol fast blue diluted in 95% EtOH overnight while incubated at 56°C. Excessive Luxol fast blue was washed out by 95% EtOH followed by distilled water. The slides were then dipped in 0.05% lithium carbonate for 30 sec, washed with distilled water and stained with 0.1% cresyl violet for 30 sec. After washing with distilled water, the brain slides were dehydrated using serial dilutions of 95% EtOH, 100% EtOH and xylene. Finally, the brain slides were covered with mounting media and cover glass.

Dead and viable neuronal cells were counted in the cerebral cortex and dorsal hippocampus cornus ammonis (CA) 1 and 3 and dentate gyrus (DG). Viable cells were characterized by light purple cytoplasm and the appearance of the nucleus and nucleolus. Dead cells were characterized by a dark purple cell with vacuole surrounding. Cerebral cortices were captured at 100X magnification (Olympus Tg300) for 3 images of each hemisphere. Each image was 886.26×668.01 µm. Granular and pyramidal cells were counted; their sizes ranged from 5 to 30 µm. The CA 1 and 3 and DG of the dorsal hippocampus were captured at 400X magnification for 3 images of each area of interest in each hemisphere. Each image was 166.67×166.34 µm. Pyramidal cells in CA1 and CA3 with sizes 15 to 35 µm and granular cells in the DG with sizes between 9 to 25 µm, were counted. White matter area images were captured at 400X magnification for 3 images of each hemisphere. White matter density in areas of interest, such as the corpus callosum, internal capsule and optic tract were analyzed for myelinated fiber density using NIH Image J (Thong-asa and Tilokskulchai, 2014 ▶; Wakita et al., 2002 ▶).

**Statistical analysis**

All data were analyzed using a one-way analysis of variance (ANOVA) followed by Fisher’s PLSD *post hoc* test. Statistical significance was accepted when p values were less than 0.05 and data are represented as mean±standard error of mean (SEM).

## Results

**Animal body and organ weights**

In all mice, continuous administration of the vehicle or *T. triandra* leaf extract had no effect on body or organ weight (Table 1). 

**Total protein level**

**Figure 1:**
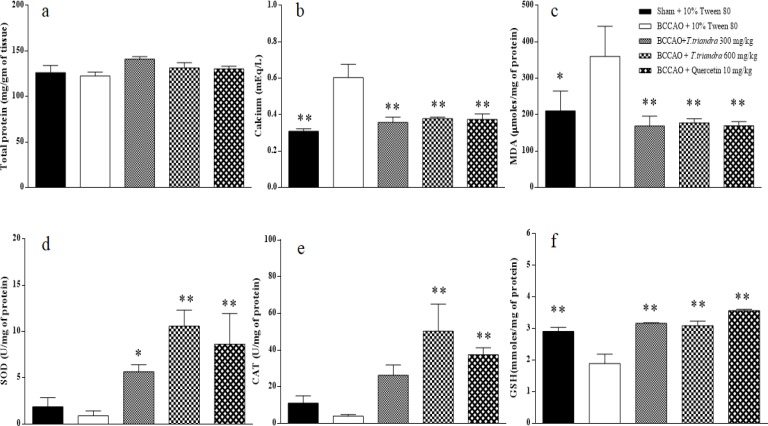
The histogram of brain tissue biochemical analysis. Total protein level (a), calcium level (b), MDA level (c), SOD level (d), CAT level (e) and GSH level (f). *p<0.05 and **p<0.01 show significant differences as compared to BCCAO + 10% Tween 80

Total brain tissue protein level was slightly reduced in the BCCAO + 10% Tween 80 group, but we found no significant difference among groups (Figure 1a).

**Calcium level**

Calcium levels increased significantly in the BCCAO + 10% Tween 80 group when compared to the Sham + 10% Tween 80 group (p=0.0003) and other BCCAO groups (i.e. *T. triandra* 300 mg/kg group (p=0.0007) and *T. triandra* 600 mg/kg (p=0.0015) group). Calcium level amelioration in the *T. triandra*-treated groups was not different from that of the quercetin 10 mg/kg group (Figure 1b).

**Malondialdehyde level**

MDA levels in the BCCAO + 10% Tween 80 group significantly increased after IR compared to the Sham + 10% Tween 80 group (p=0.0332). A comparison of MDA levels among the BCCAO groups showed a significant reduction in the BCCAO + *T. triandra* 300 mg/kg (p=0.0065), BCCAO + *T. triandra* 600 mg/kg (p=0.0085) and quercetin 10 mg/kg (p=0.0098) groups (Figure 1c). 

**Superoxide dismutase level**

Decreased SOD levels were found after IR, but there was no significance difference compared to the Sham + 10 % Tween 80 group (p=0.677). SOD levels significantly increased in the BCCAO + *T. triandra* 300, *T. triandra *600 mg/kg and quercetin 10 mg/kg groups when compared to the BCCAO + 10% Tween 80 group (p=0.042, 0.0005 and 0.005, respectively) (Figure 1d). 

**Catalase level**

CAT levels were reduced by IR, but no significance difference was found when comparing the Sham + 10% Tween 80 group with the BCCAO + 10% Tween 80 group (p=0.600). A significant CAT level increase was found only in the BCCAO + *T. triandra* 600 mg/kg (p=0.002) and BCCAO + quercetin 10 mg/kg (p=0.026) groups as compared to the BCCAO + 10% Tween 80 group (Figure 1e).

**Reduced glutathione level**

GSH levels were significantly reduced in the BCCAO + 10% Tween 80 group compared to the Sham + 10% Tween 80 group (p=0.001). GSH levels in the BCCAO + *T. triandra* 300, *T. triandra *600 mg/kg and quercetin 10 mg/kg groups significantly increased when compared to the BCCAO + 10% Tween 80 group (p=0.0002, 0.0002 and <0.0001, respectively) (Figure 1f). 

**Table 1:** Body and organ weights (mean±SEM)

**Weight (g)**	**Groups**				
	**Sham + 10% Tween 80**	**BCCAO + 10% Tween 80**	**BCCAO + ** ***T. triandra*** ** 300 mg/kg**	**BCCAO + ** ***T. triandra*** ** 600 mg/kg**	**BCCAO + quercetin 10 mg/kg**
**Body**	42.85±1.84	38.00±2.00	42.33±1.45	46.00±4.00	38.00±2.70
**Brain**	0.62±0.01	0.61±0.02	0.64±0.03	0.59±0.02	0.63±0.01
**Livers**	2.30±0.11	2.01±0.15	2.07±0.21	2.23±0.58	1.97±0.09
**Lungs**	0.26±0.01	0.31±0.03	0.29±0.06	0.32±0.05	0.28±0.01
**Stomach**	0.33±0.01	0.27±0.01	0.26±0.01	0.27±0.03	0.31±0.003
**Kidneys**	0.69±0.04	0.60±0.03	0.57±0.02	0.64±0.06	0.61±0.009
**Heart**	0.26±0.01	0.23±0.02	0.21±0.02	0.23±0.005	0.20±0.01
**Spleen**	0.11±0.01	0.09±0.01	0.07±0.007	0.07±0.01	0.07±0.009
**Testes**	0.33±0.03	0.33±0.01	0.37±0.01	0.36±0.05	0.33±0.02

**Infarction area**

**Figure 2:**
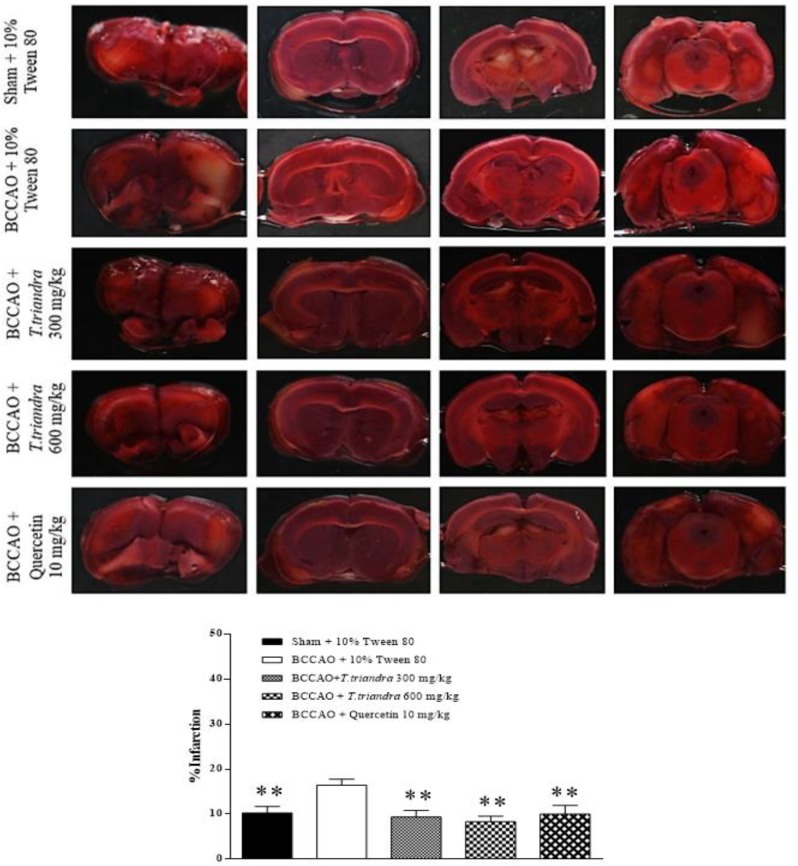
The photomicrograph of brain slides stained with TTC. The histogram shows the percentage of brain infarction (% infarction). *p<0.05 and **p<0.01 show significant differences as compared to BCCAO + 10% Tween 80

The percentage of brain infarction was significantly increased in the BCCAO + 10% Tween 80 group (p=0.008). The BCCAO + *T. triandra* 300, *T. triandra* 600 

mg/kg and quercetin 10 mg/kg groups experienced significantly decreased brain infarction compared to the BCCAO + 10% Tween 80 group (p=0.001, 0.008 and 0.003, respectively) (Figure 2). 

**Histological analysis**

The percentage of dead cells in all areas of the dorsal hippocampus significantly increased after IR induction in the BCCAO + 10% Tween 80 group (CA1; p=0.0012, CA3; p=0.0027 and DG; p=0.0023). The 

**Figure 3:**
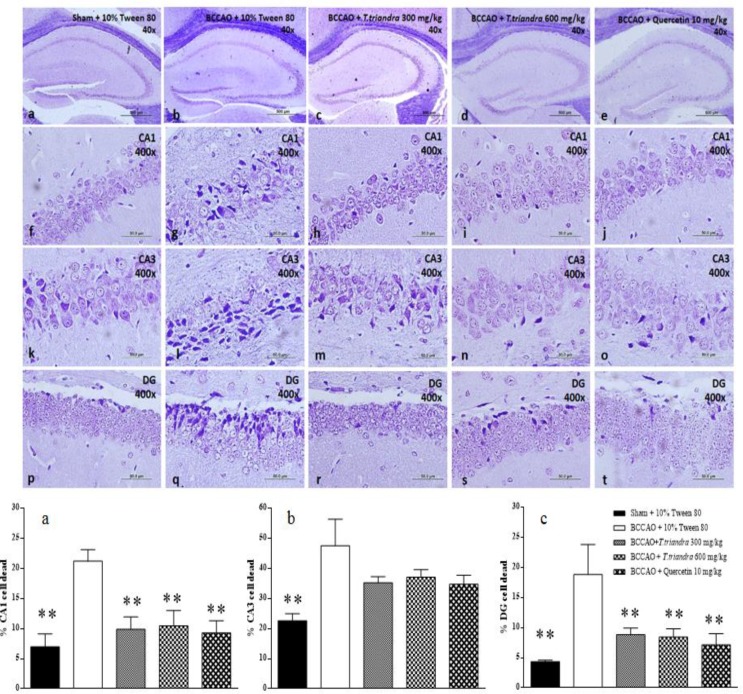
The photomicrograph of the dorsal hippocampus stained with 0.1% cresyl violet (a–e, 40X of magnification, scale bar 500 µm). The area of interest CA1 (f-j), CA3 (k-o) and DG (p-t) captured at 400X of magnification, scale bar 50 µm. Histograms show the percentage of dead cells in CA1 (a), CA3 (b) and DG (d). *p<0.05 and **p<0.01 show significant differences as compared to BCCAO + 10% Tween 80

BCCAO + *T. triandra* 300, *T. triandra* 600 mg/kg and quercetin 10 mg/kg groups had a significant reduction in the percentage of dead cells in CA1 (p=0.0052, 0.0033 and 0.0038, respectively) and DG (p=0.0187, 0.0155 and 0.0083, respectively) (Figure 3).

**Figure 4:**
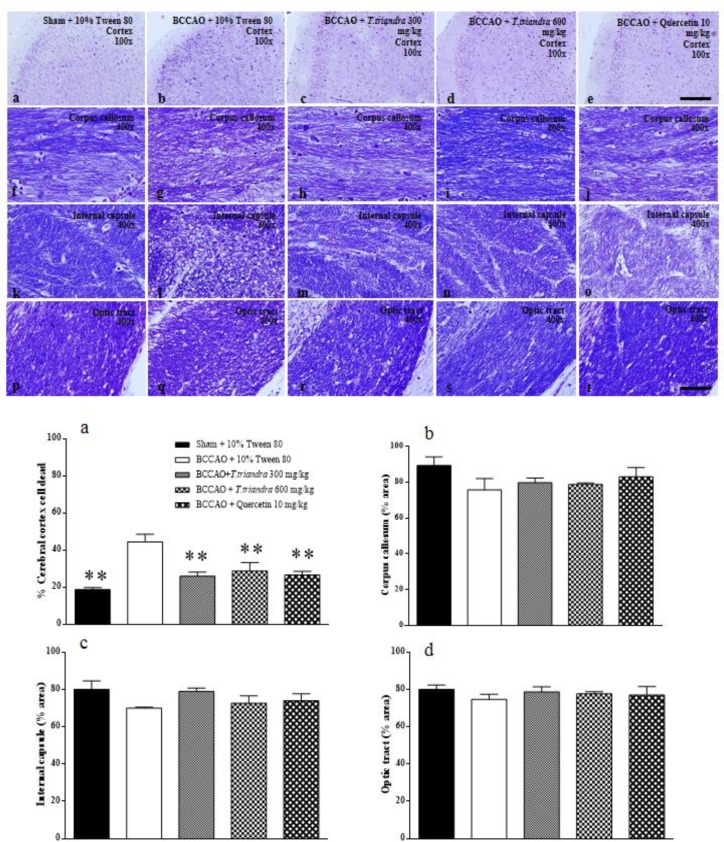
The photomicrograph of cerebral cortex stained with 0.1% cresyl violet (a–e, 100X of magnification, scale bar 200 µm). The photomicrograph of white matter area with 0.1% Luxol fast blue staining. Corpus callosum (f-j), internal capsule (k-o) and optic tract (p-t) captured at 400X of magnification, scale bar 50 µm. Histograms show the percentage of dead cells in cerebral cortex (a), white matter density (% area) in corpus callosum (b), internal capsule (d) and optic tract (d). *p<0.05 and **p<0.01 show significant differences as compared to BCCAO + 10% Tween 80

Histological analysis of the cerebral cortex (Figure 4a–e, and Histogram a) revealed a significant increase in the percentage of dead cells after IR induction (p=0.0002). Treatment with *T. triandra* at doses of 300 and 600 mg/kg and quercetin at 10 mg/kg, significantly reduced the percentage of dead cells in the cerebral cortex (p=0.0018, 0.0048 and 0.0022, respectively). 

White matter density in the corpus callosum, internal capsule and optic tract did not vary significantly among the groups (Figure 4f–t, and Histograms b–d). 

## Discussion

The present study demonstrated the neuroprotective effects of *T. triandra* leaf extract against cerebral IR injury. Treatment with *T. triandra* at doses of 300 and 600 mg/kg prevented brain oxidative stress, brain infarction and neurodegeneration in vulnerable brain areas, such as the cerebral cortex and dorsal hippocampus, similar to those induced by quercetin 10 mg/kg. Thirty minutes of BCCAO followed by 45 min of reperfusion in this study’s mice model, caused significant oxidative brain damages similar to a previous study (Raghavendra et al., 2009 ▶). IR-induced augmentation of brain tissue calcium and lipid peroxidation was represented by increased MDA levels. The mechanism of brain IR injuries, including ischemia, leads to the deprivation of high-energy phosphates such as ATP, excitotoxicity, and depolarization and augmentation of cytosolic calcium (Phillis et al., 2002 ▶). High calcium levels initiate many intracellular metabolic cascades. For example, activation of nitric oxide synthase (NOS) and the formation of NO radicals and phospholipase activation lead to membrane damage (Beckman, 1991 ▶). Lipid peroxidation is exacerbated during the reperfusion period, corresponding with overwhelming O_2_ levels that react with NO and lead to peroxynitrite (^-^ONOO) formation. As a potential free radical, ^-^ONOO initiates lipid peroxidation during the reperfusion period, which inhibits growth factor signaling and leads to apoptotic cascade   (White et al., 2000 ▶). Inhibition of the synthesis of proteins such as antioxidant enzymes during reperfusion, leads to oxidative stress (Guo et al., 2012 ▶; Mansoorali et al., 2012 ▶). 

The present study found that *T. triandra* leaf extract ameliorates oxidative stress. This was represented by brain tissue calcium and MDA reduction and a significant increase in the antioxidant enzymes SOD and CAT, as well as the reducing agent GSH. These effects involve high phenolic and flavonoid contents, especially quercetin (Boonsong et al., 2009 ▶; Phunchango et al., 2015 ▶; Singthong et al., 2014 ▶). The present study used *T. triandra* leaf extract with high antioxidant contents, including total phenolic levels of 340.21 mg GAE/g and total flavonoid levels of 231.29 mg QE/g (Thong-asa et al., 2017 ▶). Moreover, the active contents of *T. triandra* leaf extract, such as saponin, quercetin, beta-carotene, phyrol and alpha-tocopherol, are well-known antioxidant and anti-inflammatory agents (Boonsong et al., 2009 ▶). The possible survival-promoting mechanisms of *T. triandra* leaf extract during IR may include the amelioration of calcium and MDA, as well as the augmentation of the reducing agent GSH and the antioxidant enzymes CAT and SOD. These effects enhance neuronal survival from ischemic onset through the reperfusion period. Therefore, they increase neuronal endurance and may further benefit rehabilitation. 

The present study also investigated the protective effects of *T. triandra* leaf extract against IR injury in terms of brain infarction. We demonstrated that IR significantly increases brain infarction and but *T. triandra* at doses of 300 and 600 mg/kg prevents brain infarction. We confirmed this neuroprotective effect through the histological evaluation of vulnerable brain areas, such as the cerebral cortex and the dorsal hippocampus. 

The neuroprotective effects of *T. triandra* leaf extract against cerebral IR injury include antioxidation via inhibition of calcium and lipid peroxidation, as well as activation of SOD and CAT enzymes and GSH. Considering the balance between death- and survival-promoting mechanisms in IR, *T. triandra* leaf extract enhances survival-promoting mechanisms and leads to the reduction of brain vulnerability to ischemia, which may extend the therapeutic window for patients who suffer from IR. 
